# Peristomal adenocarcinoma 16 years after colorectal adenocarcinoma resection with curative intent

**DOI:** 10.1093/jscr/rjad419

**Published:** 2023-07-20

**Authors:** Kayleigh A M van Dam, Thaís T T Tweed, Bart de Vries, Henricus J Belgers

**Affiliations:** Department of Surgery, Division of Gastro-intestinal Surgery, Zuyderland Medical Center, Sittard-Geleen and Heerlen, the Netherlands; Department of Surgery, Maastricht University Medical Centre+, Maastricht, the Netherlands; Department of Pathology, Zuyderland Medical Center, Sittard-Geleen and Heerlen, the Netherlands; Department of Surgery, Division of Gastro-intestinal Surgery, Zuyderland Medical Center, Sittard-Geleen and Heerlen, the Netherlands

## Abstract

Metachronous colorectal cancer is relatively rare, occurring in 0.7–3.6% of patients diagnosed with colorectal adenocarcinoma. Cutaneous metastases are similarly a rare presentation, occurring in <6% of metastatic colorectal cancer patients. Even more rare are the cutaneous recurrences at the peristomal site. Clinically, it is difficult to distinguish between metachronous cancer and cutaneous metastases. This paper reports a case of an elderly woman presenting with a slowly progressing peristomal cutaneous lesion 16 years after surgical resection for colorectal cancer. Core punch biopsy revealed a cutaneous localization of an intestinal type of adenocarcinoma. A surgical resection of the peristomal area was carried out whereby a new colostomy was created on the contralateral side. Definite histopathological examination showed a superficially located intestinal type adenocarcinoma with extensive pagetoid spread in the epidermal surface. In conclusion, it is important to remain alert and strive for early detection for cutaneous abnormalities following colorectal cancer.

## INTRODUCTION

The standard follow-up after colorectal cancer diagnosis spans over a period of 5 years according to Dutch guidelines [1]. These guidelines consist of several diagnostic measures at certain timepoints to check for recurrences and metastases as seen in Table 1 [1]. The most common distant metastases are to the lungs or liver; however new primary colorectal tumors can also develop, such as metachronous tumors.

**Table 1:** Colorectal follow-up according to Dutch Guidelines [1]

Examination type	Year 1	Year 2	Year 3	Year 4	Year 5
CEA	Every 3–6 months	Every 3–6 months	Every 6–12 months	Every 6–12 months	Every 6–12 months
Abdominal and thoracic CT scan	After 12 months	In case of abnormal CEA levels^*^			
Colonoscopy	After 12 months OR after 3 months in case of incomplete pre-operative colonoscopy				

^*^ *CEA levels are considered abnormal in case of levels >5 ng/ml, repeating increasing levels or a doubling regarding baseline level*

Metachronous colorectal cancer is a new primary tumor occurring >6 months after the initial colorectal cancer. It is a relatively rare and easily missed presentation, occurring in 0.5–3.6% of all patients diagnosed with a colorectal adenocarcinoma [2–4]. Various pathways have been described in literature, including physical mucosal damage, direct extension or *de novo* metaplasia [2, 5]. Metachronous tumors developing at the colostomy site are even more rare with only a few cases reported [6]. Due to its low incidence, there is no standard treatment method regarding cutaneous/peristomal adenocarcinoma. However, excision is one of the preferred options when surgically feasible [7–9]. In the few cases reporting metachronous cancers at the colostomy site, the abnormality is diagnosed within 5 till 14 years after primary surgery, mostly just after the follow-up period [2, 10–12]. Varying clinical presentations are reported, such as stoma obstruction/stenosis, increased bleeding tendency and difficulty fitting the colostomy bag [2, 10–12].

Skin abnormalities at the peristomal site could also be a cutaneous metastatic lesion. Cutaneous metastasis occurs in <6% of all colorectal metastatic cancer patients [13]. The various described pathways include hematogenous or lymphatic spread, direct extension or direct implanting during surgery [14]. Regarding cutaneous metastases varying clinical morphology is reported, such as nodules, plaques and ulcers. In most of these cases the abnormality is diagnosed within 5 years after primary surgery [15–20]. The common regions are the head, chest, abdomen and the anogenital region [15, 17–21].

This case report presents an unusual presentation of a late-occurring metachronous colorectal adenocarcinoma 16 years after initial diagnosis. The purpose of this case report is to raise awareness and to promote early detection of both metachronous adenocarcinoma and cutaneous metastatic lesions at colostomy site.

## CASE DESCRIPTION

**Figure 1:**
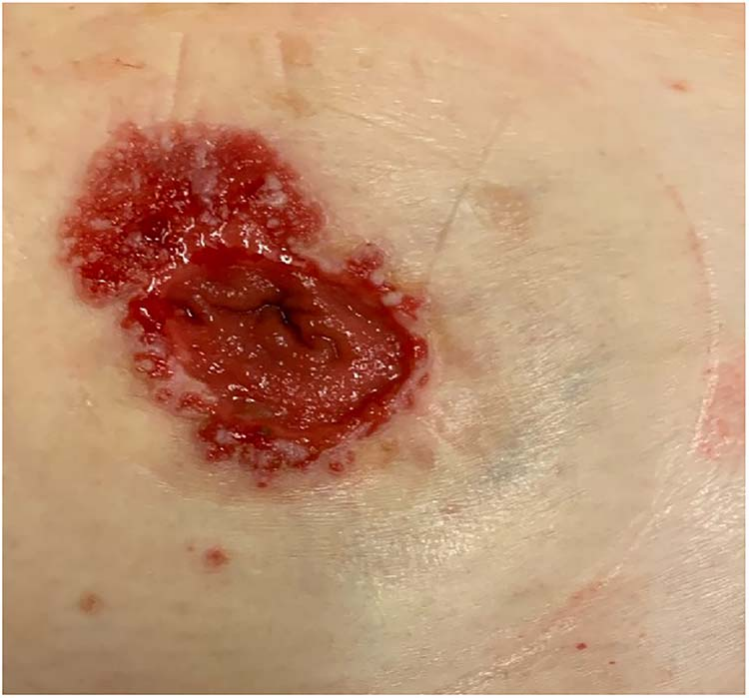
Peristomal skin abnormality.

A female patient presented to our hospital with peristomal cutaneous abnormalities (see Fig. 1). The patient was initially diagnosed with a T4N2M0 colorectal adenocarcinoma 16 years prior. At the primary diagnosis, she presented with symptoms of blood and mucus in the stool, weight loss of 6 kg within a 10 month-span and elevated carcinoembryonic antigen (CEA) levels of 19.3 ng/ml. A pelvis magnetic resonance imaging scan showed a rectal tumor over a minimum distance of 12 cm in the rectum and sigmoid junction and the diagnosis of colorectal adenocarcinoma was confirmed by rectal biopsy. The patient received neoadjuvant chemo-radiation and underwent an abdominoperineal resection. During follow-up CEA levels remained low, with an average of 1.8 ng/ml (range 0.9–2.4) during 14 outpatient visits. Colonoscopy after 1 and 5 years showed no abnormalities.

Except for the colorectal carcinoma and a bladder tumor the medical history of the patient was unremarkable, and the patient was a healthy, non-smoking woman. Initially, the peristomal cutaneous abnormality was classified as a rash and stoma granulomas. However, conservative treatment consisting of ointment and silver nitrate had no effect. A dermatologist was consulted, and further research was initiated.

## INVESTIGATIONS AND TREATMENT

**Figure 2:**
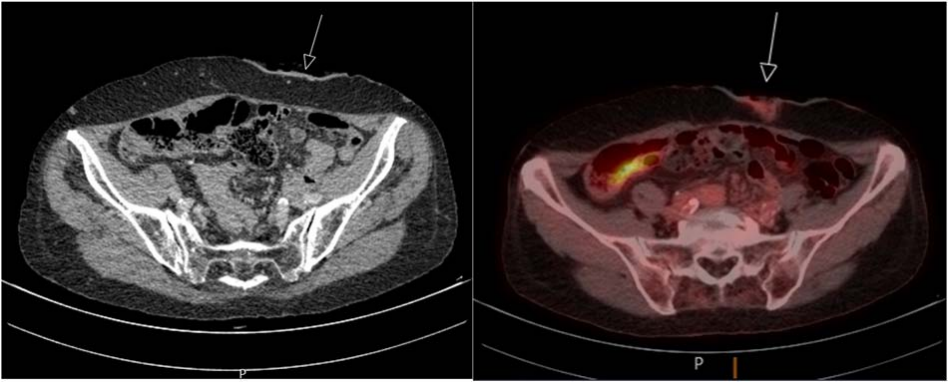
PET/CT scan showing minimal FDG stacking at the colostomy site.

A biopsy taken from the peristomal environment revealed a cutaneous located intestinal type adenocarcinoma of colorectal origin. The subsequently performed positron emission tomography–computed tomography (PET/CT) scan showed physiological intestinal stacking and minimal fluorodeoxyglucose (FDG) stacking surrounding the colostomy site (Fig. 2). In addition, a focal hypodense thyroid nodule was found. Laboratory tests showed a stable tumor marker CEA value of 2.4 ug/L compared with a last known value of 2.3 ug/L.

Multidisciplinary consultation resulted in a colostomy resection plan with margin down to the fascia due to no disease dissemination on the PET/CT scan. In addition, a new colostomy would be placed on the contralateral side. Due to the expected defect size a plastic surgeon was consulted for advice on closure technique.

Surgery was performed under general anesthesia. Upon laparoscopic inspection, there were no intra-abdominal abnormalities or signs of peritoneal implants. Adhesiolysis on the colostomy was performed and the descending colon was mobilized until enough length was created to reach the contralateral side. A new colostomy was created, and the fascia was closed with Vicryl and the skin with Monocryl. The colon was opened and attached to the skin with Monocryl transcutaneous sutures. The newly created colostomy was temporarily covered with gauze.

**Figure 3:**
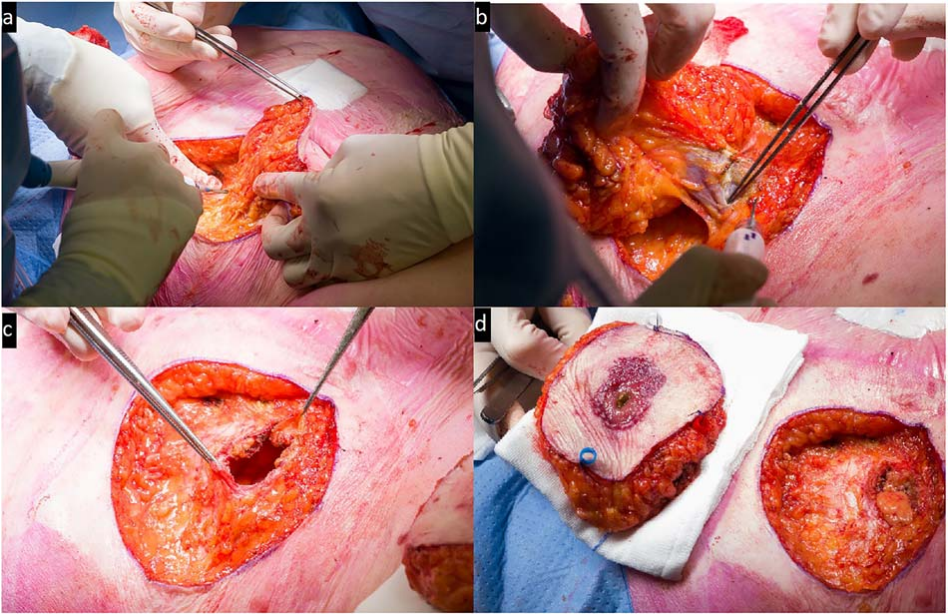
Intra-operative view: **(a)** resection of the peristomal area; **(b)** removal of the previous colostomy; **(c)** the defect including open fascia; **(d)** side-to-side resected area and the remaining defect.

Next, the old colostomy location was marked with a 3 cm margin. The area was resected in its entirety down to the fascia, resulting in a total excised area of 11 × 9 × 3 cm (Fig. 3). The resected tissue was marked with beads and sent in for histopathological examination. The fascial defect of the old colostomy was closed with PDS while the remaining fascia was closed with Vicryl. The remaining subcutaneous and cutaneous defects were closed by the plastic surgeon through transposition of the defect toward the midline due to surplus skin. This resulted in primary closure with Monocryl for the subcutis and the skin.

## FOLLOW-UP

**Figure 4:**
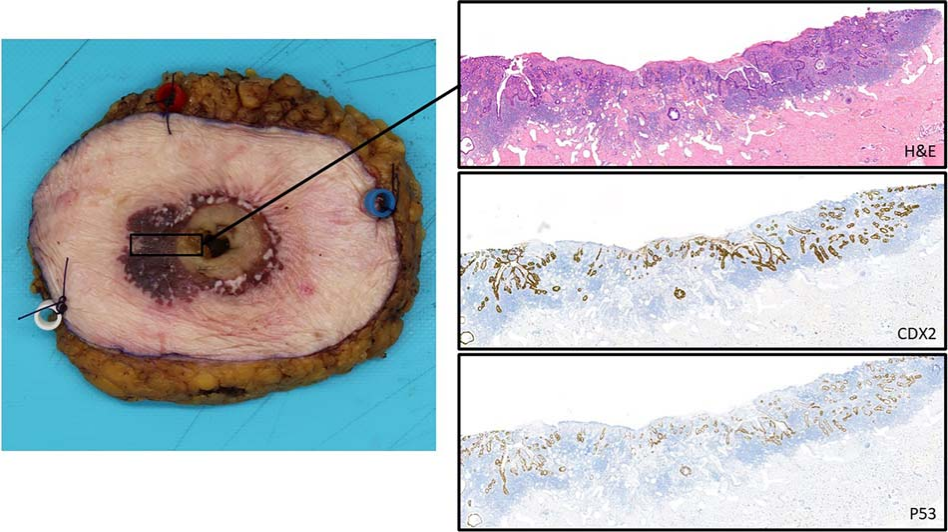
Histopathological examination showing the gross specimen and microscopic pictures; the H&E staining shows the superficial intestinal type adenocarcinoma with intra-epithelial pagetoid extension; the CDX2 staining accentuates the intestinal differentiation with pagetoid extension; the P53 staining highlights the neoplastic nature of this (intra-epithelial) lesion.

The patient was transported to the surgical ward postoperatively and was hospitalized for 2 days. The examination of the resected preparation by the pathologist confirmed a superficially invasive intestinal adenocarcinoma with extensive pagetoid spread into the epidermis. The lesion seemed to have originated from stomal mucosa at the stoma-cutaneous transition. Further pathological examination revealed superficial infiltration in the dermis (2 mm) (Fig. 4). The resection margins were tumor-free and two resected lymph nodes contained no malignant cells.

Outpatient clinic visits at 13 and 15 days postoperatively showed no abnormalities regarding wound healing. After multidisciplinary consultation only close follow-up was advised. The follow-up will be in accordance with the guidelines for colorectal carcinoma whereby the first check-up showed a CEA value of 1.8 ug/L [1].

## DISCUSSION

The patient in this report was initially diagnosed with a T4N2M0 colorectal adenocarcinoma for which she received curative treatment. During the 5-year follow-up, according to National Dutch Guidelines, no abnormalities or signs for recurrences and metastases were found. Sixteen years later, she presented with a peristomal cutaneous abnormality which was proven to be a metachronous cutaneous adenocarcinoma originated from stomal mucosa. The rash-like presentation could possibly be attributed to an early presentation with no mass formation yet, compared with the mass formation as is seen in most of the reported cases [6]. From the possible mechanisms of pathogenesis physical mucosal damage or *de novo* metaplasia/dysplasia seem to be the most likely cause in our patient. The lesion was resected, and the standard 5-year follow-up was set in motion.

In general, the recommendations for follow-up are up to 5 years as 90% of recurrences occur in this period [1, 22]. Our patient is an example of late-occurring metachronous adenocarcinoma and instigates the discussion of prolonged follow-up after colorectal cancer [22–24]. Two recent studies show late recurrence rates in the 5 till 10-year period of 6.1% in total and 2.1% for rectal and 10.7% colonic cancer [23, 24]. Regarding cutaneous metastases the literature shows that these usually occur within 5 years of the initial diagnosis, often even within 2 years [15–20].

As metachronous colorectal tumors develop >6 months after a previous colorectal tumor, arguably the same colorectal cancer standard of care and follow-up can be applied [4]. However, there are no standard recommendations for care and follow-up in case of peristomal cutaneous lesions following colorectal cancer. The diagnostic measurements in most cases consist of biopsy of the abnormality. The presence of single of multiple lesions can be an indicator for possible treatment options. In case of multiple metastatic lesions there is often chosen for palliative treatment due to the poor prognosis, either with chemo- or radiotherapy [8, 18]. With a single lesion several treatment options have been used in the literature, namely radiotherapy and surgical excision [17, 21]. For the closure of the defect, primary closure or a flap may be used depending on the size. Factors influencing the decision regarding closure technique are inability to fully clear the tumor, infected skin and predisposition to impaired vascular supply.

One of the most important prognostic factors is the time interval between initial colorectal cancer and a recurrence. Patients with an early recurrence, within 2 years after primary surgery, have a lower patient survival (32 vs 77 months) and a 5-year survival rate (34.7% vs 78.8%) compared with patients with a late recurrence [25, 26]. Therefore, early detection remains important.
